# *CCR5* editing by *Staphylococcus aureus* Cas9 in human primary CD4^+^ T cells and hematopoietic stem/progenitor cells promotes HIV-1 resistance and CD4^+^ T cell enrichment in humanized mice

**DOI:** 10.1186/s12977-019-0477-y

**Published:** 2019-06-11

**Authors:** Qiaoqiao Xiao, Shuliang Chen, Qiankun Wang, Zhepeng Liu, Shuai Liu, Huan Deng, Wei Hou, Dongcheng Wu, Yong Xiong, Jiafu Li, Deyin Guo

**Affiliations:** 1 Laboratory of Medical Virology, School of Medicine, Sun Yat-sen University, Zhongshan Erlu 74, Yuexiu District, Guangzhou, 510080 People’s Republic of China; 2 Institute of Medical Virology, School of Basic Medical Sciences, Wuhan University, Wuhan, 430071 People’s Republic of China; 3 Department of Pathology, Zhongnan Hospital, Wuhan University, Wuhan, 430071 People’s Republic of China

**Keywords:** CCR5, HIV-1, CRISPR/SaCas9, Primary CD4^+^ T cells, Human CD34^+^ hematopoietic stem/progenitor cells

## Abstract

**Background:**

The chemokine receptor CCR5, which belongs to the superfamily of G protein-coupled receptors, is the major co-receptor for HIV-1 entry. Individuals with a homozygous *CCR5Δ32* mutation have a long lasting and increased resistance to HIV-1 infection. Therefore, *CCR5* represents an optimal target for HIV-1/AIDS gene therapy. The CRISPR/Cas9 system has been developed as one of the most efficacious gene editing tools in mammalian cells and the small-sized version from *Staphylococcus aureus* (SaCas9) has an advantage of easier delivery compared to the most commonly used version from *Streptococcus pyogenes* Cas9 (SpCas9).

**Results:**

Here, we demonstrated that *CCR5* could be specifically and efficiently edited by CRISPR/SaCas9 together with two sgRNAs, which were identified through a screening of 13 sgRNAs. Disruption of CCR5 expression by lentiviral vector-mediated CRISPR/SaCas9 led to increased resistance against HIV-1 infection in human primary CD4^+^ T cells. Moreover, humanized mice engrafted with *CCR5*-disrupted CD4^+^ T cells showed selective survival and enrichment when challenged with CCR5 (R5)-tropic HIV-1 in comparison to mock-treated CD4^+^ T cells. We also observed *CCR5* could be targeted by CRISPR/SaCas9 in human CD34^+^ hematopoietic stem/progenitor cells without obvious differentiation deficiencies.

**Conclusions:**

This work provides an alternative approach to disrupt human *CCR5* by CRISPR/SaCas9 for a potential gene therapy strategy against HIV-1/AIDS.

**Electronic supplementary material:**

The online version of this article (10.1186/s12977-019-0477-y) contains supplementary material, which is available to authorized users.

## Background

Although the clinical application of highly active antiretroviral therapy (HAART) effectively inhibits HIV-1 replication and prolongs lifespan of the patients with acquired immunodeficiency syndrome (AIDS), it cannot eradicate the latent reservoir of the virus [1, 2]. Additionally, HAART has limitations due to its high cost, drug resistance, requirement for long-term adherence to treatment, and side effects such as toxicity and even immune dysfunction [3–7]. Therefore, it is necessary to look for more effective approaches to eliminate HIV-1 proviral DNA in the latent reservoir of infected individuals and pursue a cure for HIV-1/AIDS patients. In recent decades, gene therapy has been developed as a new strategy for improving the health of patients with genetic diseases, such as hemophilia [8], β-thalassemia [9] and other monogenic diseases [10]. The strategies involve using nucleases for specific gene editing to cure disease. In previous studies, zinc finger nuclease (ZFN) and transcription activator-like effector nuclease (TALEN) were the two special nucleases which could recognize the genomic editing locus by protein–DNA interaction [11, 12] and both of them had been applied in generating resistance to HIV-1/AIDS infection [13–15]. However, these two strategies have several limitations in application, including low editing efficiency and time-consuming production. In recent years, clustered regularly interspaced short palindromic repeats (CRISPR) in complex with CRISPR-associated protein 9 (Cas9) has been widely used for gene editing in mammalian cells. The Cas9 proteins derived from *Streptococcus pyogenes* (SpCas9) or *Staphylococcus aureus* (SaCas9), combined with a single small guide RNA (sgRNA) and type II CRISPR system from bacteria, can recognize and cleave DNA loci followed by a 5′-protospacer adjacent motif (PAM) sequence of NGG and NNGRRT, respectively [16–19]. DNA cleavage induces double-stranded DNA breaks (DSBs), which are repaired via error-prone non-homologous end joining (NHEJ) or homologous recombination (HR) in eukaryotes, resulting in deletions and insertions (indels) or substitution in the target sequences of the genome [19].

HIV-1 enters into cells via initial binding of gp120 envelope protein to the cellular receptor CD4 [20], followed by one of the two chemokine co-receptor CCR5 or CXCR4 [21, 22]. CCR5 is the major co-receptor for CCR5 (R5)-tropic HIV-1 [23], while CXCR4 is used as the co-receptor for CXCR4 (X4)-tropic HIV-1 that appears in about half of late-stage infections [24]. Previous studies showed that individuals with the naturally occurring *CCR5Δ32* mutation were resistant to HIV-1 infection [25, 26]. Further, the ‘Berlin patient’, an individual with acute myelocytic leukemia (AML) and HIV-1/AIDS, lived free of HIV-1 infection after receiving bone marrow from a donor with the *CCR5Δ32* genotype, suggesting a key role for *CCR5* in HIV-1 infection [27, 28]. In addition, a recent report about the ‘London patient’ with Hodgkin’s lymphoma provides evidence for HIV-1 remission by *CCR5Δ32* hematopoietic stem-cell (HSC) transplantation [29]. Thus, it is important to develop HIV cure strategies based on preventing or disrupting the expression of CCR5 co-receptor. Previous reports suggested that specific targeting of *CCR5* in human autologous CD4^+^ T cells by ZFN, TALEN or CRISPR/SpCas9  protected against HIV-1 infection [13, 15, 30–32]. Additionally, efficient ablation of *CCR5* had been achieved in human hematopoietic stem/progenitor cells and induced pluripotent stem cells by CRISPR/SpCas9 [33–36]. In recent years, a smaller SaCas9 has attracted more attention for its effective gene editing ability and ease of delivery. The adeno-associated virus (AAV)-SaCas9 system has been successfully applied in gene knock-in and knock-out studies, suggesting the possibility for SaCas9 used in HIV-1/AIDS gene therapy researches [18, 37–40]. Indeed, previous researches had shown that disruption of co-receptor CXCR4 and HIV-1 provirus by SaCas9/gRNAs promoted human primary CD4^+^ T cells and Jurkat T cells resistance to HIV-1 infection [41, 42]. It had also been reported that excision of HIV-1 provirus by SaCas9 and multiplex sgRNAs had been achieved in humanized mice models [40]. Therefore, the CRISPR/SaCas9 system is considered as a beneficial and effective gene editing tool with potential to be an HIV-1/AIDS treatment strategy.

In this study, we identified two sgRNAs that could guide SaCas9 specifically and efficiently to target *CCR5*. By using a lentiviral vector for delivery, we observed efficient editing of *CCR5* in primary human CD4^+^ T cells, leading to cell resistance to HIV-1 infection. Moreover, we showed survival and enrichment of *CCR5*-disrupted CD4^+^ T cells in humanized mice during R5-tropic HIV-1 infection. We also demonstrated that SaCas9/sgRNA induced *CCR5* editing in CD34^+^ hematopoietic stem/progenitor cells without obvious differentiation deficiencies. Together, our data suggest that *CCR5* can be effectively edited by CRISPR/SaCas9 with selected target sgRNAs and a small-sized SaCas9, which may provide an alternative approach for *CCR5* disruption in HIV-1/AIDS gene therapy.

## Results

### RNA-guided SaCas9 nuclease mediates efficient disruption of *CCR5* to protect TZM-bl cells from R5-tropic HIV-1 infection

**Fig. 1:**
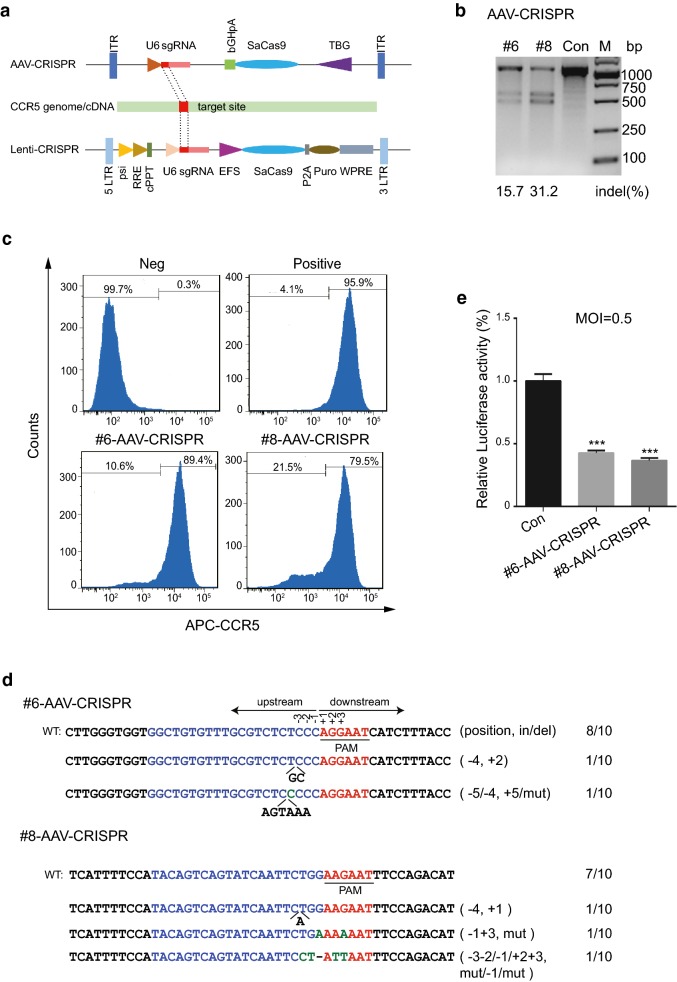
Efficient disruption of *CCR5* in TZM-b1 cells by AAV-CRISPR/SaCas9 defenses HIV-1 infection. **a** The diagram of AAV-CRISPR-SaCas9 and Lenti-CRISPR-SaCas9 vector composition and insert sites of *CCR5*-sgRNA. **b** T7E1 assay analysis of disruption efficiency in TZM-bl cells transfected with AAV-CRISPR/SaCas9-sgRNA-#6, #8 or control. The indel percentage was calculated using Image J software. **c** Flow cytometry detection of CCR5 expression on cell surface. The TZM-bl cells transfected with AAV-SaCas9/sgRNA-#6, #8 or control were stained with APC-conjugated CCR5 antibody and analyzed by flow cytometry. The unstained control-transfected TZM-bl cells and stained control-transfected TZM-bl cells were treated as negative and positive controls respectively. The data showed on the top of each peak were the percentage of CCR5 negative and positive cells. **d** DNA sequences of *CCR5* target sites in the TZM-bl cells mediated by sgRNA-#6 and #8. PCR products amplification from genomic DNA were cloned into T-vector and performed Sanger sequencing. The red sequences indicate the PAM sequences; the blue sequences are marked as the targeted sequences; the green bases in the targeted sequences are mutations; insertions and deletions are indicated with (+) and (−) respectively. N/N shows the ratio of mutations or wild type (WT) in the all sequenced clones. **e** Disruption of *CCR5* in TZM-bl cells resistance to HIV-1 infection. TZM-bl cells edited by sgRNA-#6, #8 or control were infected with R5-tropic HIV-1_YU2_ (MOI = 0.5) and analyzed by luciferase reporter assay. Data were analyzed by unpaired *t*-test and error bars showed the mean ± SEM of three independent experiments (*p < 0.05; **p < 0.01; ***p < 0.001)

To identify effective target sites, we used an online tool (http://crispr.cos.uni-heidelberg.de/) to design 13 sgRNAs with the PAM sequence of 5′-NNGRRT-3′ to target the open reading frame (ORF) of *CCR5*, in addition, we used an effective sgRNA of *CXCR4* [41] as a negative control for targeting of *CCR5* (Additional file 1: Fig. S1; Additional file 4: Table S1). To select efficient sgRNAs, we first inserted all designed target DNA (referred to as sgRNAs) into an AAV-CRISPR-SaCas9 (PX601) plasmid (Fig. 1a). We then tested the efficiency of the AAV-CRISPR/SaCa9 system and targeting of the *CCR5* gene by the sgRNAs in HeLa cells. Three days after transfection of AAV-SaCas9/sgRNA, we conducted T7 endonuclease 1 (T7E1) assays, which could detect and cleave mismatched DNA. The result showed that the 1054 bp PCR products from the cell genome could be edited by sgRNA-#2, #6, # 8 and #11 delivered by AAV-CRISPR-SaCas9, while the negative sgRNA and control showed no cleavage (Additional file 2: Fig. S2). We also found that sgRNA-#6 and #8 had much higher gene editing efficiency than others (Additional file 2: Fig. S2).

To further confirm the editing efficiency of sgRNA-#6 and #8 in an HIV-1 reporter cell line, we then transfected AAV-SaCas9/sgRNAs or control into the TZM-bl cells, an HIV-1-susceptible cell line originally adapted from HeLa cells expressing human CD4, CCR5, and an HIV-1 LTR-driven luciferase reporter [43, 44]. We conducted a T7E1 assay 3 days post-transfection. Our results showed that PCR products of the *CCR5* gene in TZM-bl cells could be cleaved into two fragments by both sgRNAs, suggesting that sgRNA-#6 and #8 efficiently induced *CCR5* disruption in TZM-bl cells (Fig. 1b). To further analyze whether the sgRNAs could disrupt CCR5 expression in TZM-bl cells, we measured the protein levels of CCR5 on the cell surface 3 days post-transfection by flow cytometry. Results indicated a 10.6% and 21.5% reduction of CCR5 expression upon treatment with sgRNA-#6 and #8, respectively (Fig. 1c). We then inserted the two PCR products of the target loci into a T-vector and determined the indels by DNA sequencing. The results showed that sgRNA-#6 and #8 induced indels and mutations in *CCR5* gene (Fig. 1d). Next, we determined whether the disruption of CCR5 expression by selected sgRNA-guided SaCas9 cleavage could resist HIV-1 infection. We transfected TZM-bl cells with AAV-SaCas9/sgRNA-#6, #8 or control and then infected the modified cells with R5-tropic HIV-1_YU2_ strain. Three days post-infection, the cells were collected and a luciferase reporter assay was performed to analyze the infection efficiency. We observed that HIV-1 infection levels in *CCR5*-edited cells were significantly reduced compared to the control-treated cells (Fig. 1e).

**Fig. 2:**
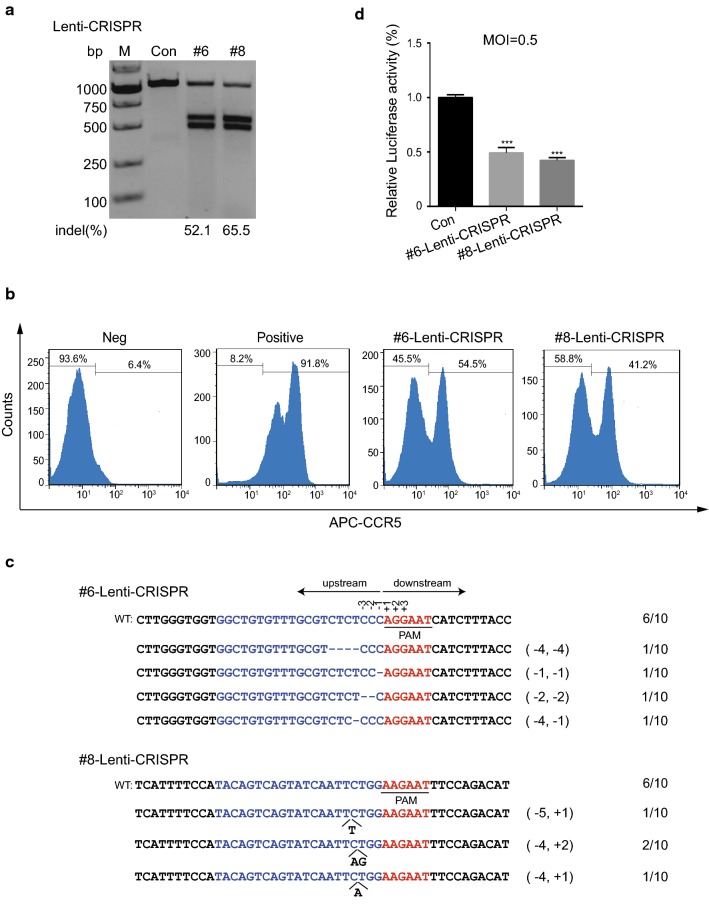
LentiCRISPR/SaCas9 mediated cleavage of *CCR5* in TZM-bl cells against HIV-1 infection. **a** LentiCRISPR/SaCas9 editing of the *CCR5* gene in TZM-bl cells. sgRNA-#6, #8 or control were transfected into TZM-bl cells delivered by lentiviral vector. Three days post transduction, genomic DNA was extracted and T7E1 assay was conducted. **b** Detection of *CCR5* expression in TZM-bl cells by flow cytometry. **c** Sanger sequencing of *CCR5* target loci in the TZM-bl cells modified by LentiCRISPR-SaCas9/sgRNA. **d** Disruption of *CCR5* in TZM-bl cells could render cell resistance to HIV-1 infection. The cells were transduced with LentiCRISPR-SaCas9/sgRNA-#6, #8 or control. Then the modified cells were infected with HIV-1_YU2_ (MOI = 0.5). Luciferase reporter assay was performed to analyze infection efficiency. Data were analyzed by unpaired *t*-test and error bars showed the mean ± SEM of three independent experiments (*p < 0.05; **p < 0.01; ***p < 0.001)

As sgRNA-#6 and sgRNA-#8 functioned efficiently in the CRISPR/SaCas9 system delivered by AAV vector, we wanted to know whether they could be delivered by lentiviral vector. In order to construct the Lenti-CRISPR/SaCas9 system, we replaced SpCas9 in the lentiCRISPR-v2 plasmid with the PCR-amplified SaCas9 from the PX601 plasmid. DNA targets of sgRNA-#6 and sgRNA-#8 were then inserted into the Lenti-CRISPR/SaCas9 recombinant vector (Fig. 1a). After analyzing the system by sequencing, we transfected Lenti-SaCas9/sgRNA-#6, #8 or control into TZM-bl cells. The T7E1 assay of the cell genome indicated that PCR products of *CCR5* gene could be cleaved by sgRNA-#6 and sgRNA-#8 efficiently (Fig. 2a). Further, flow cytometry analysis demonstrated that the reduction of CCR5 expression mediated by sgRNA-#6 and #8 could reach 45.5% and 58.8% respectively compared to control (Fig. 2b). Lastly, results of DNA sequencing (Fig. 2c) and the HIV-1 challenge assay (Fig. 2d) showed that Lenti-SaCas9/sgRNA-#6 and #8 could achieve highly efficient disruption of *CCR5* and thus confer cell resistance against HIV-1 infection. All together, these results demonstrated that disruption of *CCR5* in HIV-1 reporter cell lines by SaCas9/sgRNA-#6 and #8 protected cells from R5-tropic HIV-1 infection and sgRNAs delivered by Lenti-CRISPR/SaCas9 had higher efficiency than AAV- CRISPR/SaCas9.

### Disruption of *CCR5* in Jurkat T cells by LentiCRISPR/SaCas9 confers resistance to R5-tropic HIV-1 infection

**Fig. 3:**
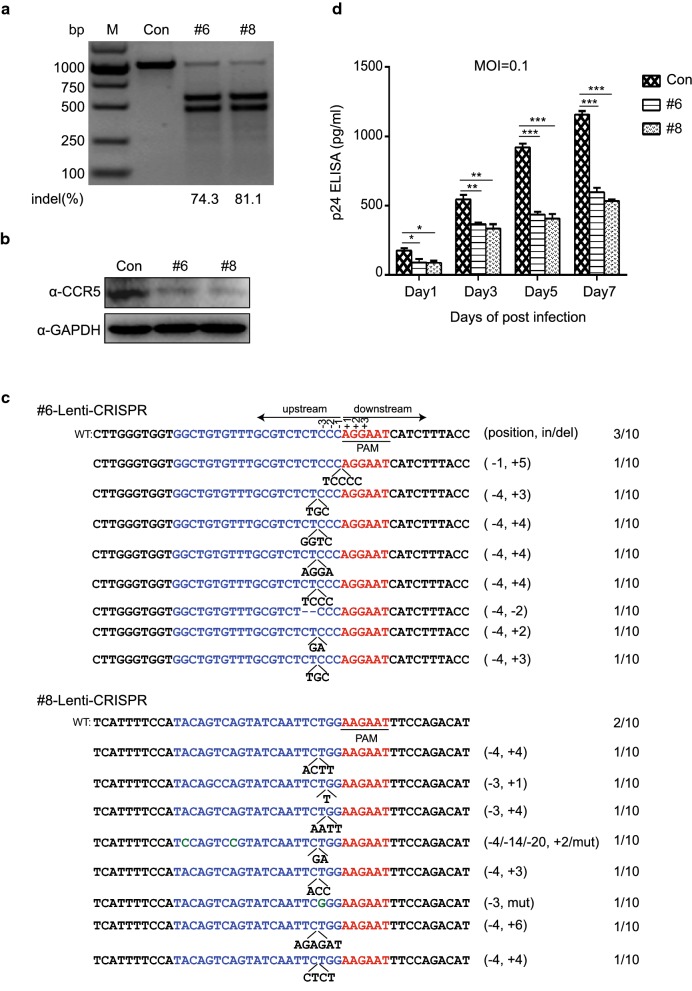
Disruption of *CCR5* in Jurkat T cells protects cells from HIV-1 infection via LentiCRISPR/SaCas9. **a** Mutation of *CCR5* in Jurkat T cells was detected by T7E1 assay. Jurkat T cells were transduced with SaCas9/sgRNA-#6, #8 or control lentivirus with MOI of 40. After 3 days selection by puromycin, the cells were harvested for T7E1 assay. **b** Western blotting assessment of CCR5 expression on SaCas9/sgRNA-lentivirus edited Jurkat T cells. *CCR5*-modified or control Jurkat T cells in **a** were collected for western blotting assay with anti-CCR5 and anti-GAPDH antibodies. **c** DNA sequencing of *CCR5* gene target loci in the modified Jurkat T cells. **d** The *CCR5*-sgRNA transduced Jurkat T cells attenuated HIV-1_YU2_ infection. The SaCas9/sgRNA-#6, #8 or control-treated Jurkat T cells were infected with R5-tropic HIV-1_YU2_ (MOI = 0.1). After 1, 3, 5, 7 days infection, cell culture medium was collected for p24 ELISA assay. Data were analyzed by unpaired *t*-test and error bars showed the mean ± SEM of three independent experiments (*p < 0.05; **p < 0.01; ***p < 0.001)

To recapitulate the *CCR5* gene editing results obtained in TZM-bl cells, we tested whether sgRNA-#6 and #8 together with SaCas9 could disrupt CCR5 expression in a CD4^+^ T cell line. After several unsuccessful attempts to disrupt *CCR5* in Jurkat T cells by AAV-CRISPR/SaCas9, we conducted lentiviral vector-mediated transduction of SaCas9/sgRNA-#6, #8 or control into Jurkat T cells to disrupt the *CCR5* gene. Three days after transduction, T7E1 assay was performed to determine the disruption efficiency of *CCR5*. The result showed that sgRNA-#6 and #8 could induce *CCR5* mutation in Jurkat T cells with high efficiency (Fig. 3a). Consistently, immunoblotting of the transduced cells indicated that the expression of CCR5 on the surface of Jurkat T cells was markedly reduced upon treatment with sgRNA-#6 and #8 compared to control (Fig. 3b). In a parallel assay, DNA sequencing showed indel mutations in the *CCR5* targeting sites (Fig. 3c), suggesting that the *CCR5* gene in Jurkat T cells could be efficiently edited by lentiviral-mediated SaCas9/sgRNA delivery.

To determine whether disruption of *CCR5* in Jurkat T cells by Lenti-SaCas9/sgRNA could result in increased resistance to HIV-1 infection, Jurkat T cells with *CCR5* mutation induced by sgRNA-#6, #8 or control were challenged with R5-tropic HIV-1_YU2_. At 1, 3, 5, and 7 days post-infection, we collected cellular supernatant and performed an ELISA assay for HIV-1 p24. The results indicated that the level of p24 in *CCR5*-edited Jurkat T cells decreased significantly compared to that in control (Fig. 3d). These results demonstrated that sgRNA-#6 and #8 delivered by lentiviral vector could efficiently induce SaCas9 cleavage of the *CCR5* gene in Jurkat T cells, leading to increased cell resistance to HIV-1 infection.

### LentiCRISPR/SaCas9-mediated *CCR5* gene editing in human primary CD4^+^ T cells confers resistance to R5-tropic HIV-1 infection

**Fig. 4:**
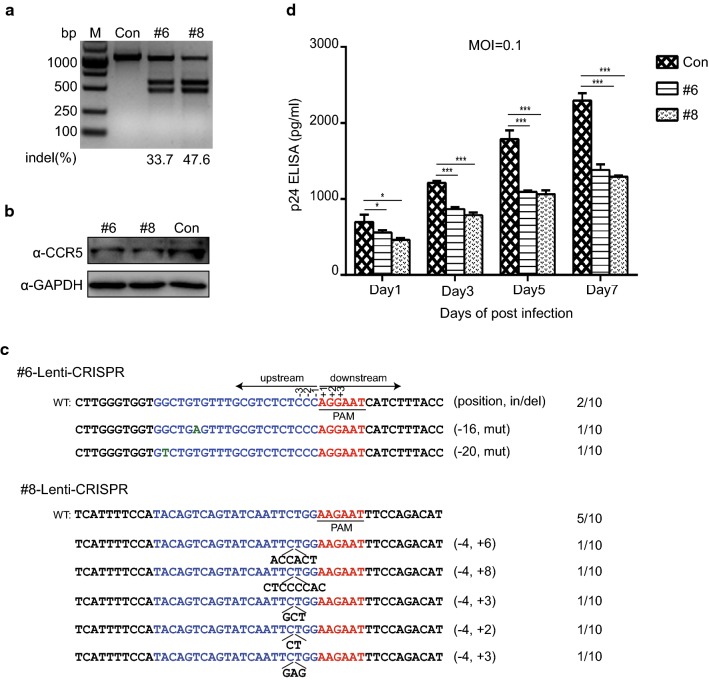
Ablation of *CCR5* by LentiCRISPR/SaCas9 in primary human CD4^+^ T cells confers HIV-1 resistance. **a** Disruption of *CCR5* in primary CD4^+^ T cells by LentiCRISPR/SaCas9. The primary CD4^+^ T cells were transduced with LentiCRISPR-SaCas9/sgRNA-sgRNA-#6, #8 or control with MOI of 100. After 72 h transduction, cells were harvested for T7E1 assay. **b** Detection of CCR5 expression in primary CD4^+^ T cells modified by LentiCRISPR/SaCas9-sgRNA-#6. #8 or control. Modified CD4^+^ T cells were lysed and analyzed by western blotting with anti-CCR5 and anti-GAPDH antibodies. **c** Sanger sequencing of *CCR5* gene target loci in modified primary CD4^+^ T cells. **d** Ablation of *CCR5* in CD4^+^ T cells resistance to HIV-1 infection. The CD4^+^ T cells edited by SaCas9/sgRNA-#6, #8 or control were treated with HIV-1_YU2_ (MOI = 0.1) for 8 h and then cell culture medium was changed with fresh medium. The supernatant was collected at the setting time points for p24 ELISA assay. Data were analyzed by unpaired *t*-test and error bars showed the mean ± SEM of three independent experiments (*p < 0.05; **p < 0.01; ***p < 0.001)

After successfully disrupting *CCR5* in both an HIV-1 report cell line and a CD4^+^ T cell line, we next attempted to deliver LentiCRISPR/SaCa9 combined with sgRNA-#6, #8 or control plasmid into human primary CD4^+^ T cells to disrupt CCR5 expression. Human primary CD4^+^ T cells isolated from healthy donors were transduced with packaged lentivirus with an MOI of 100. Three days post-transduction, we performed the T7E1 assay and found that both sgRNA-#6 and #8 could induce *CCR5* editing in primary human CD4^+^ T cells (Fig. 4a). However, the gene editing efficiency was lower than that in Jurkat T cells, which may be due to the low lentiviral transduction efficiency in primary CD4^+^ T cells [31]. Immunoblotting showed that the expression of CCR5 decreased in sgRNA-#6 and #8 modified CD4^+^ T cells compared with control (Fig. 4b). To further confirm the disruption of *CCR5* by SaCas9/sgRNA-#6 and #8 in human CD4^+^ T cells, we sequenced 10 random PCR products in the T-vector and found indels and mutations in the target region (Fig. 4c). To evaluate whether the modification of *CCR5* in primary human CD4^+^ T cells by CRISPR/SaCas9 could render the cells resistant to HIV-1 infection, we infected the CD4^+^ T cells with HIV-1_YU2_ and then cultured these cells for 7 days. The assessment of virus by measuring the HIV-1 p24 in cell culture medium at days 1, 3, 5, and 7 post-infection, which demonstrated a decrease in the p24 level over time compared to control (Fig. 4d). These data indicated that *CCR5*-edited in human primary CD4^+^ T cells by LentiCRISPR/SaCas9 could inhibit R5-tropic HIV-1 infection.

### *CCR5*-modified human primary CD4^+^ T cells have survival advantages after challenge with HIV-1 in humanized mice

**Fig. 5:**
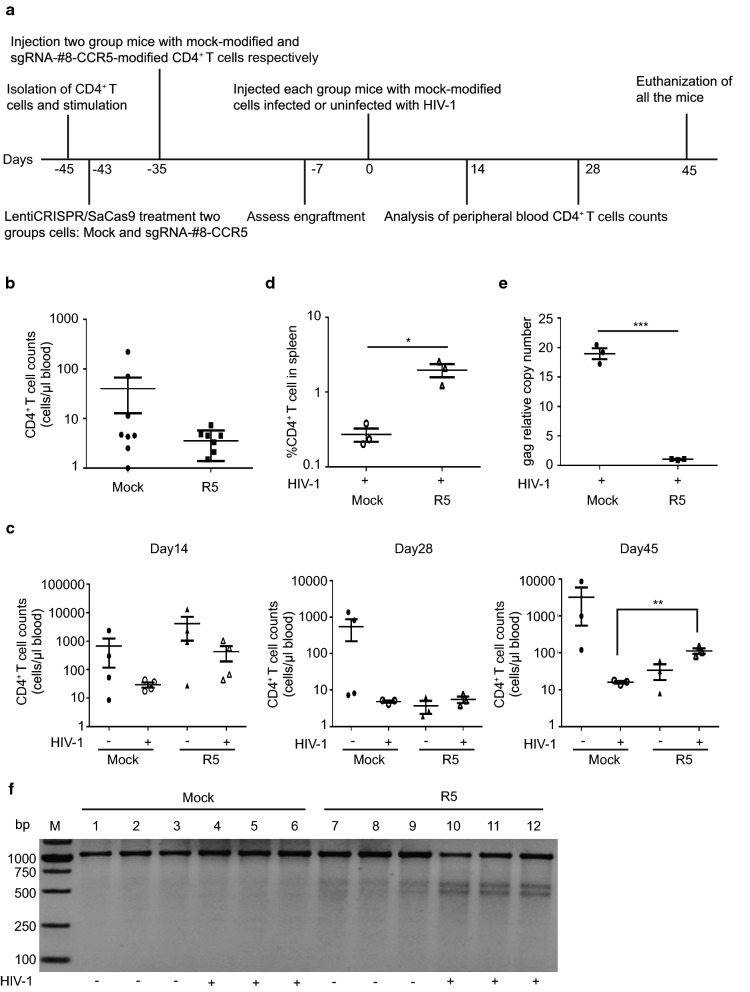
CC*R5* disruption via LentiCRISPR/SaCas9 renders CD4^+^ T cells survival from HIV-1 infection in vivo. **a** Scheme of humanized mice experiment. Experiment schedule and process were showed at each time point. 16 mice were divided into two groups randomly and each group received mock-modified or SaCas9/sgRNA-#8-*CCR5*-modified human CD4^+^ T cells. **b** Assess engraftment of all the mice post injection by flow cytometry to calculate CD4^+^ T cell counts. **c** Analysis of CD4^+^ T cell counts after infection with HIV-1. CD4^+^ T cells counts were calculated at 14, 28, 45 days post-infection by flow cytometry. **d** Measure the proportion of human CD4^+^ T cells in spleens of HIV-1 infected mice after euthanasia. **e** The relative copy numbers of gag gene in CD4^+^ T cells of spleens in **d**. **f** *CCR5* alleles mutation in CD4^+^ T cells from humanized mice. The human CD4^+^ T lymphocytes from spleens were conducted for genomic DNA extraction and T7E1 assay after 45 days infection. Mutation were showed in *CCR5*-modified CD4^+^ T cells in mice. Data were analyzed by unpaired *t*-test. Error bars showed the mean ± SEM (*p < 0.05; **p < 0.01; ***p < 0.001)

Immunodeficient mice are commonly used to model HIV-1 infection in vivo [33, 34, 45]. As we demonstrated the efficient disruption of *CCR5* by SaCas9/sgRNA in vitro, we wanted to evaluate whether CRISPR/SaCas9-mediated disruption of *CCR5* could protect primary CD4^+^ T cells against HIV-1 infection in vivo. We chose the most efficient sgRNA (#8) and injected two groups of NOD-Prkdc^em26Cd52^Il2rg^em26Cd22^/Nju (NCG) humanized mice with mock-modified human CD4^+^ T cells or LentiCRISPR-SaCas9/sgRNA-#8-modified CD4^+^ T cells (n = 8 per group) (Fig. 5a). The human CD4^+^ T cell counts in the peripheral blood of each mouse were evaluated by flow cytometry 28 days post-injection to assess engraftment. 7 days after assessing engraftment, we injected and infected half of the mice (n = 4) in each group with or without HIV-1_YU2_ (Fig. 5a).

We monitored the counts of CD4^+^ T cells in each group at different time points to assess the effect of HIV-1 infection in cells modified with CRISPR/SaCas9-sgRNA-#8 in vivo (Fig. 5a). Before HIV-1 infection, the cell counts in the group with mock-modified cells were higher than that with *R5*-modified cells (Fig. 5b). This slight engraftment difference may be donor-specific, as shown in a previous study [34]. However, in the R5-HIV-1 infection group, the difference in cell counts between mock-modified and *R5*-modified group became more obvious with time (Fig. 5c). 14 days post-infection, *CCR5*-modified cells in R5-HIV-1 infected group were ~ten-fold higher in number than mock-modified cells in R5-HIV-1 infected group (Fig. 5c, left panel). 28 days post-infection, however, we did not observe marked differences in CD4^+^ T cell counts with HIV-1 infection in mock group compared to the *R5*-modified group (Fig. 5c, middle panel), which may be due to the killing of unmodified CD4^+^ T cell by HIV-1 infection in *R5*-modified group. 45 days post-infection, CD4^+^ T cell counts of the *R5*-modified, HIV-1-infected group were about eight-fold higher than that of the mock-modified, HIV-1-infected group, and the difference was statistically significant (Fig. 5c, right panel). As expected, the majority of mice injected with *R5*-modified or mock-modified CD4^+^ T cells showed xenogeneic graft versus host disease (XGVHD) with clinical features of hair loss and dermatitis, which often appear between 47 and 52 days post-engraftment [46]. We sacrificed the mice that had developed XGVHD and the final numbers of the mice that remained were equivalent in modified and unmodified groups, indicating that *R5*-SaCas9 itself might not alter CD4^+^ T cell function and have few side effects in vivo.

Furthermore, we determined the percentage of human CD4^+^ T cells in the spleen after euthanasia of the HIV-infected mice. The mice that received *R5*-modified CD4^+^ T cells had about 4% human CD4^+^ T cells of total cells in spleens. In contrast, human CD4^+^ T cells were almost undetectable in the spleens of the mice infused with mock-modified CD4^+^ T cells (Fig. 5d). We also performed real-time PCR assay to detect relative copy number of the HIV-1 gag gene in human CD4^+^ T cells in the spleens, and the results demonstrated a lower copy number of gag normalized to β-globin in R5-modified CD4^+^ T cells infected with HIV-1 compared to that of control (Fig. 5e). Importantly, T7E1 assay of the *CCR5* gene in CD4^+^ T cells from the spleens showed the mutation in *CCR5*-modified CD4^+^ T cells with or without HIV-1 infection (Fig. 5f), which was indicative of successful modification and engraftment of CD4^+^ T cells in humanized mice. In addition, *R5*-modified cells were enriched in HIV-1-infected mice compared with uninfected mice (Fig. 5f). Therefore, *CCR5*-modified CD4^+^ T cells by SaCas9 showed resistance and enrichment in humanized mice.

### *CCR5* disruption in human CD34^+^ HSPCs via lentiviral vector expressing SaCas9/sgRNA

**Fig. 6:**
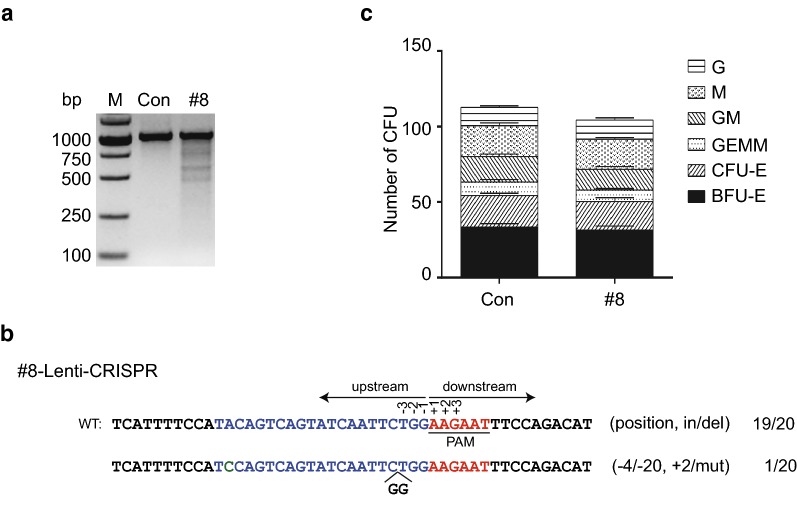
*CCR5*-editing in the human hematopoietic stem cells by LentiCRISPR/SaCas9 maintains potential multilineage ability. **a** *CCR5* gene editing by LentiCRISPR/SaCas9-sgRNA-*#*8 or control in CD34^+^ HSPCs analyzed via T7E1 assay. **b** Sanger sequencing of *CCR5* gene target loci in modified primary CD34^+^ HSPCs. **c** Numbers of colony types generated from untreated and *CCR5*-modified CD34^+^ HSPCs by CFU assay. Data were analyzed by unpaired *t*-test and error bars showed the mean ± SEM of three independent experiments

Human CD34^+^ HSPCs are a significant tool for gene therapy of some hereditary genetic disorders such as hematological diseases [47] because of their ability to generate a hematopoietic system. Disruption of *CCR5* in human CD34^+^ HSPCs by ZFN can provide long-term antiviral effects [48]. Researchers also successfully used CRISPR/SaCas9 to generate a SOX2 reporter in a human induced pluripotent stem cell line [49]. Since the LentiCRISPR/SaCas9-mediated disruption of *CCR5* in human primary CD4^+^ T cells was effective in increasing HIV-1 resistance in vitro and in vivo, we next determined whether LentiCRISPR/SaCas9 disruption of *CCR5* might have the same effect in human CD34^+^ HSPCs. We isolated human CD34^+^ HSPCs from umbilical cord blood and cultured the cells with SCF, Flt-3L and TPO. LentiCRISPR-SaCas9/sgRNA-#8 or control were transfected into human CD34^+^ HSPCs by nucleofection, and the T7E1 assay was performed to confirm disruption of *CCR5* at 72 h post-transfection. The results indicated that sgRNA-#8 induced *CCR5* gene editing in human CD34^+^ HSPCs (Fig. 6a) and DNA sequencing demonstrated deletion and mutation of the *CCR5* gene (Fig. 6b). The colony-forming unit (CFU) assay is a standard protocol to evaluate the normal differentiation potential of stem cells [33, 36]. To determine the phenotype of HSPCs development, we conducted CFU assays of non-treated and *CCR5*-modified CD34^+^ HSPCs. The numbers and types of colonies formed by control and *CCR5*-modified cells suggested that differentiation potential of HSPCs was not affected by CRISPR/SaCas9-mediated gene editing (Fig. 6c). Due to the small number of human CD34^+^ HSPCs, however, we did not conduct in vivo assays in mice with *R5*-modified human CD34^+^ HSPCs.

### CRISPR/SaCas9-mediated *CCR5* disruption is highly specific and non-toxic to cells

The type II CRISPR/Cas9 system has been widely used in recent years for various studies. Although it is a convenient and efficient approach, it may have potential off-target effects that could limit its clinical utility [50]. To test the potential off-target cleavage mediated by SaCas9/sgRNA, we compared sgRNA-#6 and #8 target sequences against the human genome to determine the potential off-target binding sites by an online search tool (http://www.rgenome.net/cas-offinder/) [51]. 10 potential off-target sites were screened (Additional file 5: Table S2) and about 800-bp PCR products from Jurkat T cell genomic DNA were tested by T7E1 assay. We did not identify indels in these potential off-target sites in Jurkat T cells edited by CRISPR/SaCa9 (Additional file 3: Fig. S3a, b), indicating the combination of sgRNA-#6 and #8 with CRISPR/SaCa9 exhibited high specificity in our experiments. When a DSB in DNA is induced, the p53 binding protein 1 (53BP1) can be recruited to the DSB site to mediate NHEJ and facilitate the repair response [30, 52]. Thus, we can quantify the number of genome-wide DSBs induced by CRISPR/SaCas9 through detection of 53BP1 foci in the nucleus. The genomic integrity of Jurkat T cells was assessed 2 days post-transduction with Lenti-SaCas9/sgRNA-#6 and #8 by observing and counting the number of 53BP1 foci per nucleus via immunostaining. Compared with etoposide-treated positive control cells, the number of 53BP1 foci per nucleus of the SaCas9/sgRNA-treated cells and untreated cells was much lower (Additional file 3: Fig. S3c). Otherwise, the MTT assay results of CRISPR/SaCas9-treated and untreated Jurkat T cells cultured in low serum medium showed no toxicity at different time points when cells were under stress (Additional file 3: Fig. S3d).

## Discussion

Traditional antiretroviral therapy for the treatment of HIV-1/AIDS maintains undetectable levels of virus replication but cannot eradicate the proviral reservoir. Throughout the course of treatment, the HIV-1 provirus remains hidden but can be activated after cessation of therapy, which is followed by productive infection and severe disease progression [2, 7, 53]. It is impossible to cure HIV-1/AIDS by drug treatment alone. In addition, antiretroviral therapy has potential side-effects and is quite expensive in some under-developed countries, making it unaffordable as a long-term treatment [3, 4, 54]. Therefore, gene therapy may be a potential alternative approach to control HIV-1 replication and cure HIV-1/AIDS in the future [55, 56].

A small number of people with a 32-bp deletion of the *CCR5* gene (*CCR5Δ32*) have shown to be resistant to HIV-1 infection [57–59], and transplantation of allogeneic bone marrow with homozygous *CCR5Δ32* to AIDS patients has shown to be a functional cure with few side effects [29, 60]. From this evidence, the chemokine receptor CCR5 represents an optimal target for HIV-1/AIDS therapy. With limited availability of *CCR5Δ32*-homozygous donors and the potential for immunological rejection, direct disruption of *CCR5* by gene editing is needed to advance HIV-1/AIDS gene therapy [32, 34, 61, 62]. ZFN-mediated disruption of *CCR5* has shown efficacy in human CD4^+^ T cells and has been applied in a phase I clinical trial [13]. Earlier gene therapy approaches such as ZFN and TALEN have potential drawbacks and limitations such as low efficiency and high off-target effects. However, the type II CRISPR/Cas9 system has been developed rapidly in recent years as a powerful gene editing tool. It has the characteristics of convenience, high editing efficacy, and low off-target effects in mammalian cells [31, 32, 63–65]. CD4^+^ T cells in which *CXCR4* was disrupted by CRISPR/SpCas9 or SaCas9 showed HIV-1 resistance [41, 66]. Here, we showed that CRISPR/SaCas9 combined with a sgRNA to target the *CCR5* in human primary CD4^+^ T cells inhibited HIV-1 infection. Moreover, *CCR5*-modified CD4^+^ T cells engrafted into humanized mice exhibited a significant survival and enrichment advantage after R5-tropic HIV-1 infection compared to unmodified cells. This indicates that HIV-1 infection may place selective pressure on *CCR5*-modified CD4^+^ T cell survival. Due to the development of XGVHD in the HIV-1-infected NCG mice, whether the enriched *CCR5*-disrupted CD4^+^ T cell population could confer long-term resistance to HIV-1 infection was not assessed in our humanized mice study. In addition, the *CCR5* gene could also be disrupted by CRISPR/SaCas9 in CD34^+^ HSPCs without deficiencies in differentiation. Therefore, CRISPR/SaCas9-mediated disruption of *CCR5* may have the potential for application in HIV-1/AIDS therapy.

Although the CRISPR/Cas9 system is a powerful genome editing technology and has multiple advantages compared to other gene editing tools [67], the potential for off-target effects must be taken into consideration prior to clinical trials [17]. The tolerance of mismatches in the target sites by SpCas9/sgRNA has been observed in a previous study [68]. In terms of the *CCR5* gene, off-target sites may exist at another C–C chemokin receptor, *CCR2,* or other genes [69]. Intriguingly, the efficient disruption of *CCR5* via lentivirus expressing spCas9/sgRNA have shown negligible off-target effects in CD4^+^ T cells [32] and in CD34^+^ HSPCs [33]. Unlike the CRISPR/SpCas9 system which has a three-base PAM sequence, CRISPR/SaCas9 has a PAM sequence that consists of six bases. This longer PAM sequence may improve on-target recognition and reduce off-target rates. In our study, we also assessed potential off-target effects of the CRISPR/SaCas9 system with *CCR5* sgRNA, and our data showed no detectable off-target editing at the selected potential off-target sites. The specificity of CRISPR/SaCas9 activity was further supported by direct staining for 53BP1 foci induced by DSB in the nucleus, which was used for the detection of cleavage at the most similar putative off-target sites in the genome. All the results suggest that ablation of *CCR5* via the CRISPR/SaCas9 system may be a safe alternative approach for generation of HIV-1 resistance in human CD4^+^ T cells and CD34^+^ HSPCs, however, there are also potential drawbacks in targeting *CCR5* alone in HIV-1/AIDS gene therapy. Disruption of the *CCR5* co-receptor may pressure the R5-tropic HIV-1 strain to use another co-receptor, *CXCR4,* to replicate in cells [70]. In addition, disruption of CCR5 expression alone cannot cure the patients infected with R4-tropic HIV-1 as well as the patients infected with both R4- and R5-tropic HIV-1. To overcome this problem, researches on simultaneous disruption of both *CXCR4* and *CCR5* genes by ZFNs and CRISPR/SpCas9 in primary human CD4^+^ T cells had been conducted, and such strategies could protect cells from both R4- and R5-tropic HIV-1 infection [34, 71, 72]. The CRISPR/SaCas9 system developed in this work can be used in future studies to target both *CXCR4* and *CCR5* for broad-spectrum resistance against various HIV strains.

In our study, we chose two highly-efficient sgRNAs screened from thirteen sgRNAs in cell lines. But the efficiencies of *CCR5* disruption in primary human CD4^+^ T cells and CD34^+^ HSPCs are lower than that in cell lines. This may be determined by many factors such as the specificity of sgRNA between cell lines and primary cells [33, 73] and inefficient delivery [31]. As shown in previous study, efficient delivery of CRISPR/Cas9 components into primary cells remains a major challenge for *CCR5* editing [31]. The AAV vector is a safe and efficient vector in gene editing [18]. As the size of SaCas9 is smaller than commonly used SpCas9, the SaCas9/sgRNA-*CCR5* system can be compatible with AAV vector [18, 41, 74]. In our study, we have difficulty in transducing sgRNA/SaCas9 delivered by AAV vectors into primary cells. Moreover, previous studies had found that inactivation of gene by AAV-SaCas9 remains a challenge in primary human cells due to issues such as special serotype and low transduction efficiency [75, 76]. Our successful delivery of SaCas9/sgRNA by lentivirus may show promise for the improvement of AAV-delivered SaCas9/sgRNA to disrupt *CCR5* in HIV-1 gene therapy. Additionally, we showed that LentiCRISPR/SaCas9-mediated disruption of *CCR5* was effective in CD34^+^ HSPCs, and whether engraftment of *CCR5*-edited CD34^+^ HSPCs into humanized mouse resistance to HIV-1 infection requires further exploration.

## Conclusions

In summary, our study demonstrated that disruption of *CCR5* using CRISPR/SaCas9 delivered by lentivirus in human primary CD4^+^ T cells resulted in T-cell prevention of HIV-1 infection and enrichment in humanized mice. The selective survival and enrichment of *CCR5*-modified CD4^+^ T cells in humanized mice challenged with HIV-1 may provide the reference for reconstitute immune function in individuals with HIV-1/AIDS. Moreover, CRISPR/SaCas9-mediated *CCR5* editing in CD34^+^ HSPCs has no effect on cell differentiation, which may point to a safe application in transplantation of allogeneic bone marrow. Overall, by using a combination of small-sized SaCas9 and novel target sites of *CCR5*, this work provides an alternative approach to specifically and efficiently disrupt *CCR5* in human cells and may offer a new choice for HIV-1/AIDS gene therapy in the future.

## Methods

### Cell lines, cell culture and transfection

HeLa cells and TZM-bl cells were maintained in DMEM (Gibco) and Jurkat T cells were maintained in RPMI 1640 (HyClone) as described in previously [77]. HeLa cells and TZM-bl cells were planted in 12-well plates and transfected with 1.0 µg AAV-CRISPR/SaCas9-sgRNA or Lenti-CRISPR/Cas9-sgRNA plasmids per well by Lipofectamine 2000 (Life Technologies) according to its instructions.

### Construction of adeno-associated viral vector and lentiviral vector expressing CRISPR/SaCas9-sgRNA

The sgRNA targets were designed by online tool (http://crispr.cos.uni-heidelberg.de/). The target DNA were synthesized, annealed and inserted into PX601 plasmid (Addgene #61591) digested by *BsaI* (Fermentas) to generate the recombinant AAV vector expressing CRISPR/SaCas9-sgRNA. To construct lentiviral vector expressing SaCas9 and sgRNA, we firstly amplified SaCas9 from PX601 by PCR and inserted it into lentiCRISPR-v2 plasmid (Addgene #52961) to replace SpCas9. Then we inserted all these sgRNA targets into LentiCRISPR/SaCas9 digested with *BsmbI* (Fermentas). All adeno-associated viral and lentiviral recombinant vectors expressing SaCas9 and sgRNA were confirmed by sequencing. The oligonucleotides used for target *CCR5* in the study were showed in Additional file 4: Table S1.

### Isolation of human primary CD4^+^ T cells and CD34^+^ hematopoietic stem/progenitor cells

All human samples handling and experimental procedures were approved by the Experimental Ethics Committee of Wuhan University. The human whole blood samples were obtained from Wuhan Blood Center (Wuhan, China) donated by healthy people. Then we separated the peripheral blood mononuclear cells (PBMCs) by centrifugation at 200*g* for 15 min with Ficoll-Paque (BD) from the whole blood. For further separation and purification of human primary CD4^+^ T cells, we used CD4^+^ T cell isolation Kit (Miltenyi Biotech) according to the manufacturer’s instructions. CD4^+^ T cells were maintained in RPMI 1640 medium (HyClone) supplemented with 10% FBS (Gibco), 1% penicillin/streptomycin (HyClone) and human interleukin-2 (IL-2) (20 ng/ml, Peprotech) at 37 °C with 5% CO_2_. CD4^+^ T cells were stimulated in anti-CD3/anti-CD28-coated (BioLegend) culture dishes before transduction [78].

The human umbilical cord blood samples were collected from Zhongnan Hospital of Wuhan University (Wuhan, China) or Wuhan Hamilton Biotechnology (Wuhan, China). The human CD34^+^ HSPCs were isolated from umbilical cord blood by CD34^+^ MicroBead Kit (MACS, Miltenyi Biotec) according to its manufacturer’s instructions. The isolated CD34^+^ HSPCs were maintain in Stemspan serum-free medium II (STEMCELL Technologies) supplemented with cytokines including recombinant human stem cell factor (SCF; 100 ng/mL, PeproTech), recombinant human fms-related tyrosine kinase 3 ligand (Flt-3L; 100 ng/mL, PeproTech) and recombinant human thrombopoietin (TPO; 100 ng/mL, PeproTech).

### Nucleofection of plasmids to human hematopoietic stem/progenitor cells and colony-forming unit (CFU) assay

About 5 × 10^6^ human CD34^+^ HSPCs were electroporated with 3.0 µg Lenti-CRISPR-SaCas9/sgRNA or control plasmids using P3 Primary Cell 4D-Nucleofector Kit (V4XP-3024). Briefly, after centrifugation of 5 × 10^6^ cells by 200*g* for 10 min, the culture medium was discarded and the cells were washed with 1xPBS for three times. Then re-suspended the cells by 100 µl Nucleofector Solution with 3.0 µg plasmids and electroporated using a Lonza Nucleofector 4D (E0-100). After nucleofection, the mixture was immediately transferred into the pre-warmed medium carefully and cultured at 37 °C with 5% CO_2_ for 6 h before replacement with fresh medium.

For CFU assay, about 2000 non-treated and *CCR5*-modified CD34^+^ HSPCs were seeded in methylcellulose medium (MethoCult H4435 Optimum, Stem Cell Technologies) according to the manufacturer’s instructions. 14 days after incubation at 37 °C with 5% CO_2_, total clone numbers were enumerated under inverted microscope.

### Production of lentivirus and HIV-1 virus and cell transduction

The HEK293T cells were seeded in 10 cm plates overnight before transfected with 6.0 µg LentiCRISPR-SaCas9/sgRNA or LentiCRISPR-SaCas9 control plasmids, 3.0 µg pMD2.G and 4.5 µg psPAX2 plasmids combining with Polyethylenimine regent (PEI, Polysciences, Warrington, PA) and opti-MEM (Gibco) according to the manufacture’s instructions. The culture medium was collected after 3 days post transduction and filtered by 0.45 µm filter. The viral titer was tested by virus counter (Virocyt 2100) and stored in − 80 °C after aliquot. Jurkat T cells were transduced with lentivirus (MOI = 40) by centrifugation at 2000 rpm for 2 h with 8 µg/ml polybrene (Sigma) at 25 °C, then incubated for another 4 h in 37 °C with 5% CO_2_ before replacement with fresh medium. The CD4^+^ T cells were cultured in the anti-CD3 and CD8 coated plate with IL-2 in the medium for 36 h and then transduced with lentivirus (MOI = 100) just as Jurkat T cells. The transduced cells were cultured in fresh medium RPMI with 10% FBS for 3 days for further analysis. The R5-tropic HIV-1 virus (HIV-1_YU2_) was produced as previous described [71].

### T7 endonuclease 1 (T7E1) assay and DNA sequencing

To measure the efficiency of *CCR5* genomic mutation, we performed T7 endonuclease 1 (T7E1) assay [79] and sequencing analysis as previously described [66]. Briefly, according to the protocol of Blood & Cell Culture DNA Midi kit (Tiangen, China), genomic DNA was extracted from the modified cells and PCR amplification of *CCR5* gene with a set of primers (Additional file 6: Table S3**)**. The PCR products were purified by Gel Extraction Kit (Promage). Then we used 300 ng purified PCR products combined with 2 µl 10 × NEB buffer (New England BioLabs) and appropriate deionized H_2_O to make the final volume of 20 µl. The mixture was annealed to form the heteroduplexes and digested with five unites of mismatch-sensitive T7E1 (New England BioLabs) for 1 h at 37 °C [79]. The digested DNA was analyzed by 1.5% agarose gel and the editing frequency was quantified by Image J software as described previously [17]. The PCR products were also inserted into pEGM-T Easy Vector (Promega) for sequencing by a T7 primer.

### Flow cytometry and western blotting

To detect the expression of *CCR5* in cells edited by CRISPR/SaCas9, we collected the control-modified and *CCR5*-sgRNA/SaCas9 modified TZM-bl cells 3 days post transfection and washed with cold 1 × PBS for three times. Then the cells were strained with APC conjugated anti-human CCR5 antibody (Biolegend) for 30 min on ice and analyzed by flow cytometry (FACS AriaIII, BD). To count the number of CD4^+^ T cells in blood obtained from the mice in different time points, we firstly treated the blood samples by red blood lysis buffer (BD Biosciences) for 15 min, then stained the samples with PE conjugated anti-human CD4 antibody (BD Biosciences), APC conjugated anti-human CD3 antibody (BD Biosciences) and FITC conjugated anti-human CD8 antibody (BD Biosciences) for 30 min on ice. At last, all samples were analyzed by flow cytometry (FACS AriaIII, BD) and Flow Jo software (Treestar). For western blotting assay, we lysed the cells with lysis buffer containing 50 mM Tris-HCl pH = 7.4, 1% Triton X-100, 150 mM NaCl, 0.1% SDS, 1.5 Mm EDTA, 0.25% deoxycholate and protease inhibitor PMSF and cocktail (Roche Applied Science) on ice for 30 min. The lysates were centrifuged by 12,000 rpm for 10 min and the supernatants were mixed with 2 × SDS loading buffer incubation at 100 °C for 10 min. The proteins were detected by SDS-PAGE with anti-GAPDH (Proteintech) and anti-CCR5 antibodies (Proteintech).

### Luciferase reporter assay, p24 antigen ELISA and real time PCR assay

*CCR5*-modified and control-modified TZM-bl cells (5 × 10^4^) were seeded in 24-well plate one day before R5-tropic HIV-1 infection. All the cells were infected with HIV-1_YU2_ (MOI = 0.5) for 8 h and washed with 1 × PBS for three times before changing fresh medium. After 3 days infection, the TZM-b1 cells were collected and lysed with 100 µl lysis buffer (Promega). 20 µl of cell suspensions were used to measure luciferase activity by a BrightGlo Luciferase assay according to the manufacturer’s instruction (Promega). For Jurkat T cells and CD4^+^ T cells, the supernatant was collected at different days post infection and analyzed by a p24 ELISA Kit (RETRO-TEK) according to its instruction. The genomic DNA of CD4^+^ T cells in spleens were extracted and real time PCR assay was conducted to detected HIV-1 gag or human β-globin gene using SYBR Green PCR Master Mix (Invitrogen).

### Humanized mice transplantation, assessment and HIV-1 challenge in vivo

All animal experiments were performed in compliance with the National Institutes of Health guidelines for the care and use of laboratory animals. Animal handling and experimental procedures were approved by the Animal Experimental Ethics Committee of Wuhan University. Human primary CD4^+^ T cells were expanded and transduced as described above. The NOD-Prkdc^em26Cd52^Il2rg^em26Cd22^/Nju (NCG) humanized mice (10–12 weeks) were injected with human CD4^+^ T cells via caudal vein. Mice were divided into two groups randomly and each group had 8 mice received with 1 × 10^7^ mock-modified or *S*aCas9/sgRNA-CCR5 modified human CD4^+^ T cells/per mice. The animals were purchased from Nanjing Biomedical Research Institute of Nanjing University (Nanjing, China) and were maintained in a defined flora animal center at college of life sciences, Wuhan university. We evaluated the number of CD4^+^ T cells in peripheral blood from each mouse eye socket vein post-injection by flow cytometry. 35 days after transplantations, half of the mice in each group received 1 × 10^5^ cells/per infection with HIV-1_YU2_ and another half received 1 × 10^5^ cells/per without virus infection. After HIV-1_YU2_ challenge, we began to collect the whole blood at different time point for three times and measured CD4^+^ T cell counts. All the mice were conducted by euthanization at 45 days post-infection and the spleen cells were collected by mechanical trituration method and passed through a 70 μm strainer to measure CD4^+^ T cell counts. SaCas9/sgRNA-induced CCR5 mutation frequency was analyzed by T7E1 assay.

### Off-target analysis

The potential off-target sites were predicted by the online tool (http://www.rgenome.net/cas-offinder/) to look for the similar sequences with the 4-bp allowed mismatch (Additional file 5: Table  S2). Those predicted off-target sequences were amplified by PCR of about 800-bp fragment centered near the off-target sites. The LentiCRISPR/SaCas9-modified Jurkat T cell genome were as templates and primes used in the analysis were listed in Additional file 6: Table S3. T7E1 assay was used to detect the off-target mutation.

### 3-(4,5-dimethylthiazol-2-yl)-2,5-dephrnyltetra-zolium bromide (MTT) assay

5 × 10^3^ Jurkat T cells were plated in 96-well plates with 2% FBS in RPMI 1640 medium and cultured for another 0, 24, 48 and 72 h. At different time points, cells were collected for MTT assay by MTT Cell Proliferation and Cytotoxicity Aaasy Kit (Beyotime, China) according to its instruction manual. Briefly, 10 µl MTT (5 mg/ml) was added into each well and incubated at 37 °C for 4 h. Then 100 µl Formanzan solution was added into each well for dissolving. The optical density was read at 570 nm by Multiscan Spectrum (Bio-Tek).

### Immunofluorescence assay

Staining for 53BP1 in the nuclear was conducted in Jurkat T cells 2 days post-transduction. The cells were fixed with 4% paraformaldehyde for 10 min at room temperature, then washed with 1 × PBS for twice and permeated with 0.5% TritonX-100 for 5 min. After blocking the nonspecific staining with 3% BSA for 30 min, cells were incubated with anti-53BP1 rabbit polyclonal antibodies (Cell Signaling Technology) diluted by 1% BSA at 4 °C overnight. The cells were incubated with Rhodamine-conjugated secondary antibody (Thermo Scientific) at room temperature for 1 h. Slides were mounted with DAPI (Thermo Scientific) to stain cell nuclei and viewed on the immunofluorescence microscope (ZEISS).

### Statistical analysis

Statistical analysis was performed by Graph-Pad Prism 5.0 and statistical significant was calculated by unpaired *t* tests. *p < 0.05; **p < 0.01, and ***p < 0.001 represents significant differences. All experiments were repeated at least three times.

## Additional files

**Additional file 1: Fig. S1.** The schematic of *CCR5* gene targeted locus in this study. The *CCR5* gene locates at the short arm of chromosome 3 and the open reading frame (ORF) of *CCR5* is in the fourth exon with the base pair from 46,370,854 to 46,376,206 referred to GRCh38 coordinate. 13 sgRNAs were designed by protospacer adjacent motif (PAM) with 5′-NNGRRT-3′ or 3′-NNCYYA-5′ sequences.

**Additional file 2: Fig. S2.** Screening of the effective *CCR5*-sgRNAs in HeLa cells by the T7E1 assay. HeLa cells were seeded in 12-well plates and transfected with 1 µg AAV-Cas9/sgRNAs. T7E1 assay was conducted in AAV-SaCas9/sgRNA modified cells 3 days post transfection. Neg: *CXCR4*-sgRNA; Con: AAV vector only; #1–#13: AAV vector expressing SaCas9/sgRNA-#1–#13.

**Additional file 3: Fig. S3.** Off-target analysis of *CCR5*-sgRNA-#6 (a) and #8 (b) in Jurkat T cells by T7E1 assay. (c) Detection of 53BP1 localization in the cell nucleus by immunostaining and epifluorescence microscopy 2 days after Jurkat T cells were transduced with lentivirus expressing SaCas9/sgRNA. Untreated cells as a negative control and 1 µM etoposide treated cells as a positive control. The consensus scale bar was 20 µm. (d) MTT assay to measure the cell viability at 0, 24, 48 and 72 h in low serum medium after transduced with lentivirus expressing SaCas9/sgRNA for 3 days in Jurkat T cells. Data were analyzed by unpaired *t*-test and error bars showed the mean ± SEM of three independent experiments.

**Additional file 4: Table S1.** Oligonucleotides of sgRNA for targeting *CCR5* locus.

**Additional file 5: Table S2.** List of potential off-target sites.

**Additional file 6: Table S3.** Primers used in the study.

## Data Availability

All relevant data are within the paper and all data are fully available without restriction.
