# Correction to: *CCR5* editing by *Staphylococcus aureus* Cas9 in human primary CD4^+^ T cells and hematopoietic stem/progenitor cells promotes HIV-1 resistance and CD4^+^ T cell enrichment in humanized mice

**DOI:** 10.1186/s12977-019-0482-1

**Published:** 2019-07-23

**Authors:** Qiaoqiao Xiao, Shuliang Chen, Qiankun Wang, Zhepeng Liu, Shuai Liu, Huan Deng, Wei Hou, Dongcheng Wu, Yong Xiong, Jiafu Li, Deyin Guo

**Affiliations:** 1 Laboratory of Medical Virology, School of Medicine, Sun Yat-sen University, Zhongshan Erlu 74, Yuexiu District, Guangzhou, 510080 People’s Republic of China; 2 Institute of Medical Virology, School of Basic Medical Sciences, Wuhan University, Wuhan, 430071 People’s Republic of China; 3 Department of Pathology, Zhongnan Hospital, Wuhan University, Wuhan, 430071 People’s Republic of China

## Correction to: Retrovirology (2019) 16:15 10.1186/s12977-019-0477-y

Following publication of their article [1], the authors realized that they inadvertently omitted the contribution of Dr. Li Wu (Ohio State University) who commented on the manuscript at the early stage of the manuscript preparation and provided one plasmid related to this work. Therefore, the authors would like to add the following to the “Acknowledgement” of the paper through this Correction article: “We gratefully thank Dr. Li Wu for his helpful comments on the earlier version of the manuscript and for providing the plasmid for HIV Yu clone”.
