# Supplementary figures and images for: CCR5 editing by Staphylococcus aureus Cas9 in human primary CD4+ T cells and hematopoietic stem/progenitor cells promotes HIV-1 resistance and CD4+ T cell enrichment in humanized mice
Source: Retrovirology. 2019 Jun 11;16:15. doi: 10.1186/s12977-019-0477-y (PMC6560749; doi:10.1186/s12977-019-0477-y)

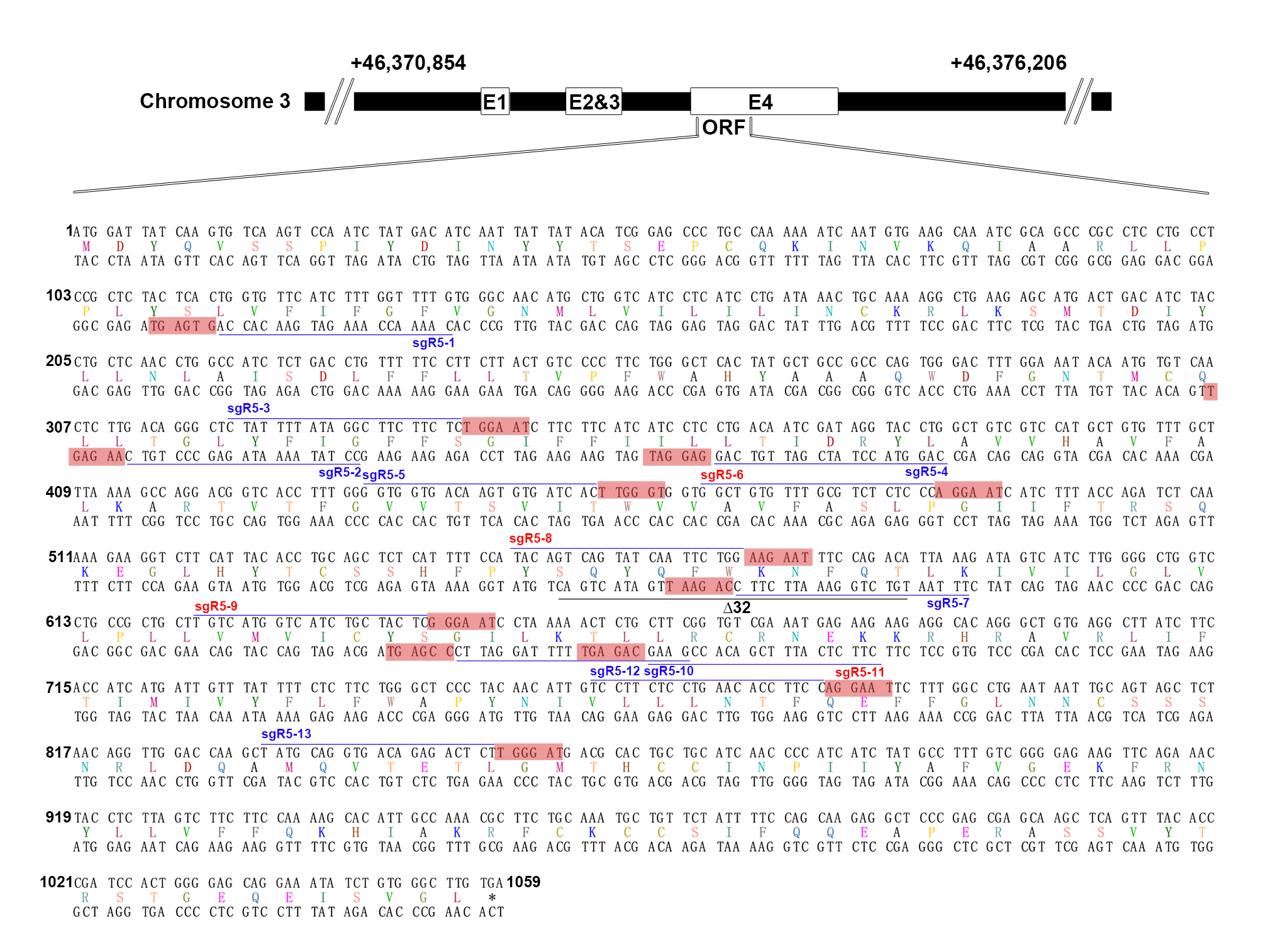

Supplement: Supplementary file 1 — Additional file 1: Fig. S1. The schematic of CCR5 gene targeted locus in this study. The CCR5 gene locates at the short arm of chromosome 3 and the open reading frame (ORF) of CCR5 is in the fourth exon with the base pair from 46,370,854 to 46,376,206 referred to GRCh38 coordinate. 13 sgRNAs were designed by protospacer adjacent motif (PAM) with 5′-NNGRRT-3′ or 3′-NNCYYA-5′ sequences. [file 12977_2019_477_MOESM1_ESM.tif]

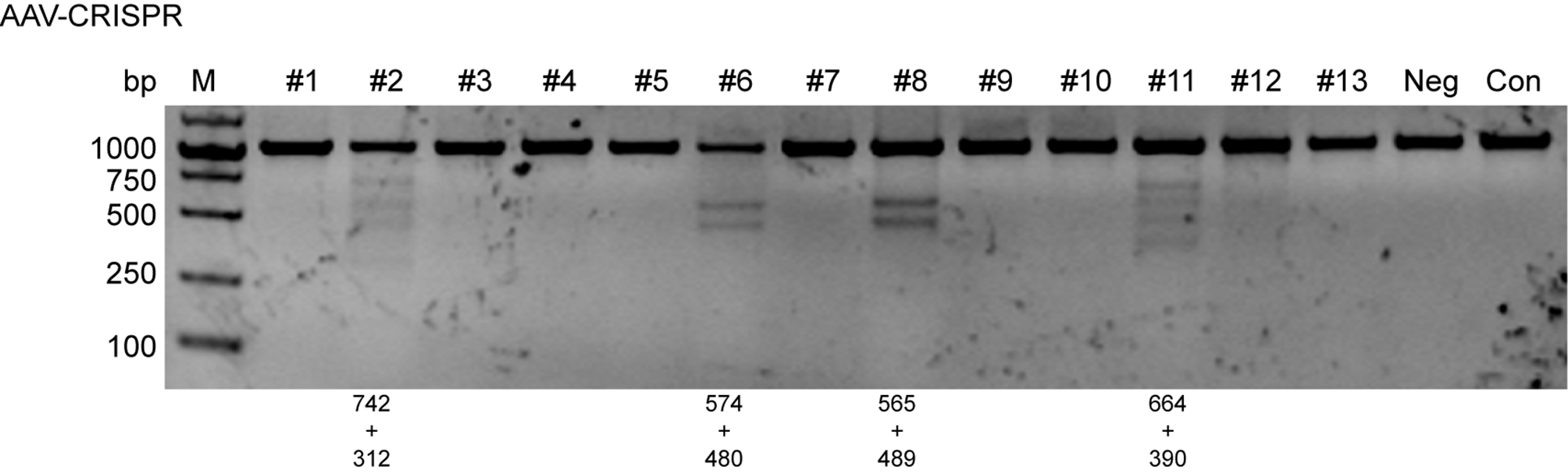

Supplement: Supplementary file 2 — Additional file 2: Fig. S2. Screening of the effective CCR5-sgRNAs in HeLa cells by the T7E1 assay. HeLa cells were seeded in 12-well plates and transfected with 1 µg AAV-Cas9/sgRNAs. T7E1 assay was conducted in AAV-SaCas9/sgRNA modified cells 3 days post transfection. Neg: CXCR4-sgRNA; Con: AAV vector only; #1–#13: AAV vector expressing SaCas9/sgRNA-#1–#13. [file 12977_2019_477_MOESM2_ESM.tif]

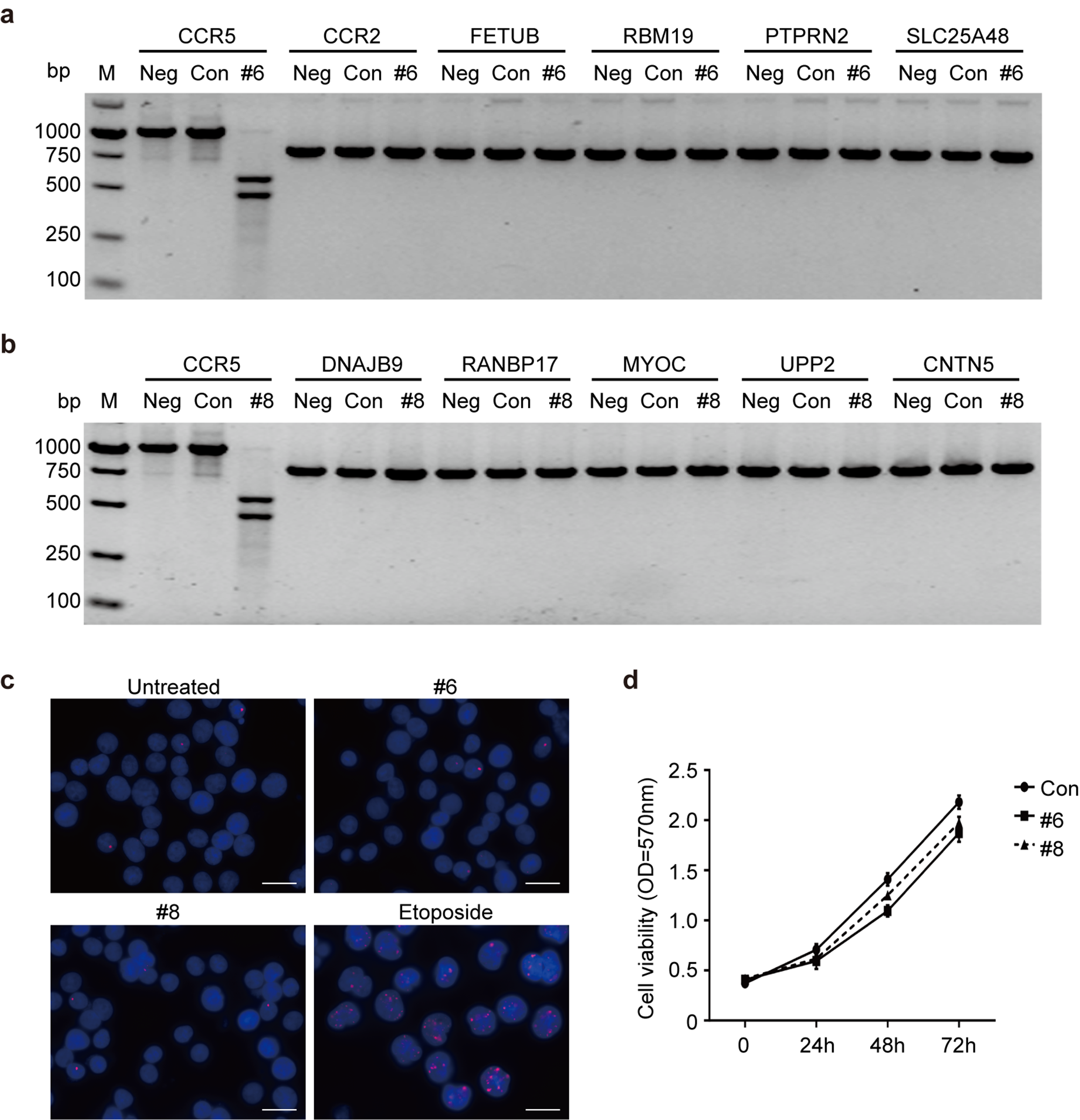

Supplement: Supplementary file 3 — Additional file 3: Fig. S3. Off-target analysis of CCR5-sgRNA-#6 (a) and #8 (b) in Jurkat T cells by T7E1 assay. (c) Detection of 53BP1 localization in the cell nucleus by immunostaining and epifluorescence microscopy 2 days after Jurkat T cells were transduced with lentivirus expressing SaCas9/sgRNA. Untreated cells as a negative control and 1 µM etoposide treated cells as a positive control. The consensus scale bar was 20 µm. (d) MTT assay to measure the cell viability at 0, 24, 48 and 72 h in low serum medium after transduced with lentivirus expressing SaCas9/sgRNA for 3 days in Jurkat T cells. Data were analyzed by unpaired t-test and error bars showed the mean ± SEM of three independent experiments. [file 12977_2019_477_MOESM3_ESM.tif]
