# Supplementary material for: CCR5 editing by Staphylococcus aureus Cas9 in human primary CD4+ T cells and hematopoietic stem/progenitor cells promotes HIV-1 resistance and CD4+ T cell enrichment in humanized mice
Source: Retrovirology. 2019 Jun 11;16:15. doi: 10.1186/s12977-019-0477-y (PMC6560749; doi:10.1186/s12977-019-0477-y)
Supplement: Supplementary file 4 — Additional file 4: Table S1. Oligonucleotides of sgRNA for targeting CCR5 locus. [file 12977_2019_477_MOESM4_ESM.docx]

Table S1. sgRNAs for targeting *CCR5* locus.

| sgRNA | target sequence on *CCR5*  (PAM: NNGRRT) | Primers sequence for target *CCR5*  (5′-3′) |
| --- | --- | --- |
| #1 | ACTCACTGGTGTTCATCTTTGGTTTTG | F: CACCGCAAAACCAAAGATGAACACCA |
|  |  | R: AAACTGGTGTTCATCTTTGGTTTTTGC |
| #2 | ACTCTTGACAGGGCTCTATTTTATAGG | F: CACCGCCTATAAAATAGAGCCCTGTC |
|  |  | R: AAACGACAGGGCTCTATTTTATAGGC |
| #3 | CTATTTTATAGGCTTCTTCTCTGGAAT | F: CACCGCTATTTTATAGGCTTCTTCTC |
|  |  | R: AAACGAGAAGAAGCCTATAAAATAGC |
| #4 | ATCCTCCTGACAATCGATAGGTACCTG | F: CACCGCAGGTACCTATCGATTGTCAG |
|  |  | R: AAACCTGACAATCGATAGGTACCTGC |
| #5 | GGTGGTGACAAGTGTGATCACTTGGGT | F: CACCGGTGGTGACAAGTGTGATCAC |
|  |  | R: AAACGTGATCACACTTGTCACCACC |
| #6 | GGCTGTGTTTGCGTCTCTCCCAGGAAT | F: CACCGGCTGTGTTTGCGTCTCTCCC |
|  |  | R: AAACGGGAGAGACGCAAACACGCC |
| #7 | ATTCTGGAAGAATTT*C*CAGACATTAAA | F: CACCGTTTAATGTCTGGAAATTCTTC |
|  |  | R: AAACGAAGAATTTCCAGACATTAAAC |
| #8 | TACAGTCAGTATCAATTCTGGAAGAAT | F: CACCGTACAGTCAGTATCAATTCTGG |
|  |  | R: AAACCCAGAATTGATACTGACTGTAC |
| #9 | TGTCATGGTCATCTGCTACTCGGGAAT | F: CACCGTGTCATGGTCATCTGCTACTC |
|  |  | R: AAACGAGTAGCAGATGACCATGACAC |
| #10 | ACTCGGGAATCCTAAAAACTCTGCTTC | F: CACCGAAGCAGAGTTTTTAGGATTC |
|  |  | R: AAACGAATCCTAAAAACTCTGCTTC |
| #11 | ACTCTGCTTCGGTGTCGAAATGAGAAG | F: CACCGCTTCTCATTTCGACACCGAAG |
|  |  | R: AAACCTTCGGTGTCGAAATGAGAAGC |
| #12 | TCCTTCTCCTGAACACCTTCCAGGAAT | F: CACCGTCCTTCTCCTGAACACCTTCC |
|  |  | R: AAACGGAAGGTGTTCAGGAGAAGGAC |
| #13 | TATGCAGGTGACAGAGACTCTTGGGAT | F: CACCGTATGCAGGTGACAGAGACTCT |
|  |  | R: AAACAGAGTCTCTGTCACCTGCATAC |
| CXCR4 | ATCCTGCCTGGTATTGTCATCCTGTCC | F: CACCGGACAGGATGACAATACCAGG  R: AAACCCTGGTATTGTCATCCTGTCC |

PAM sequences are highlighted by red. F:forward, R: reverse.
