# Supplementary material for: CCR5 editing by Staphylococcus aureus Cas9 in human primary CD4+ T cells and hematopoietic stem/progenitor cells promotes HIV-1 resistance and CD4+ T cell enrichment in humanized mice
Source: Retrovirology. 2019 Jun 11;16:15. doi: 10.1186/s12977-019-0477-y (PMC6560749; doi:10.1186/s12977-019-0477-y)
Supplement: Supplementary file 5 — Additional file 5: Table S2. List of potential off-target sites. [file 12977_2019_477_MOESM5_ESM.docx]

Table S2. List of potential off-target sites.

| sgRNA | Gene | Predicted off-target sequence  (5’-3’) | Position | Mismatches |
| --- | --- | --- | --- | --- |
| #6 | CCR2 | GGCTGTGTTTGCTTCTGTCCCAGGAAT | Chr3:46358027 | 2 |
|  | FETUB | GGCTGGGTTTTCGTCTCTACCTTGGGT | Chr3:186659885 | 3 |
|  | RBM19 | GGCTGGGTTTTCGTCTTTACCTTGGGT | Chr12:114057728 | 4 |
|  | PTPRN2 | TGCTGTGTTTGCCTCTCACCAAGGAGT | Chr7:158492589 | 4 |
|  | SLC25A48 | GGCTGAGTTTTCGTCTTTACCTTGGGT | Chr5:135735476 | 4 |
| #8 | DNAJB9 | TATAGGGAGTATAAATTCTGGTTGAAT | Chr7:108679537 | 4 |
|  | RANBP17 | TACAGTCTGAATAAAGTCTGGAAGAAT | Chr5:171176525 | 4 |
|  | MYOC | TGCAATCAGTGTCAATTCTGTTAGAAT | Chr1: 171633204 | 4 |
|  | UPP2 | TTCTGTCAGTTTCAATTCTGTTAGAAT | Chr2:157973852 | 4 |
|  | CNTN5 | CACAGTCAGTCTCAATTATGAAGGAAT | Chr11:100029666 | 4 |

Mismatched sequences are highlighted by green. PAM sequences are indicated by red. Chromosome is abbreviated as Chr.
