# Supplementary material for: CCR5 editing by Staphylococcus aureus Cas9 in human primary CD4+ T cells and hematopoietic stem/progenitor cells promotes HIV-1 resistance and CD4+ T cell enrichment in humanized mice
Source: Retrovirology. 2019 Jun 11;16:15. doi: 10.1186/s12977-019-0477-y (PMC6560749; doi:10.1186/s12977-019-0477-y)
Supplement: Supplementary file 6 — Additional file 6: Table S3. Primers used in the study. [file 12977_2019_477_MOESM6_ESM.docx]

Table S3. Primers used in this study.

| Gene name | Primer sequence  (5’-3’) | DNA size  (bp) |
| --- | --- | --- |
| CCR2 | F: AATTTGACGTGAAGCAAATTGGG | 800 |
|  | R: GATGCAGCAGTGAGTCATCCCAA |  |
| FETUB | F: GTCAGGGTACATGTACCTTTTTCTC | 801 |
|  | R: AAGATCCTGATGCAGGAAGTCCTG |  |
| RBM19 | F: CAGGGTACGTGTGCCTTTTTC | 799 |
|  | R: GGGCTTTTGGCATTTGAAGC |  |
| PTPRN2 | F: GGTTAGTCCGATGTCGTGGAAC | 800 |
|  | R: GTTGGCTTTCCTTGTGGATTTAG |  |
| SLC25A48 | F: TTAGGAGGAATCCCAGGCTGAG | 801 |
|  | R: TGCCTTTTTCTCTTTCAGACC |  |
| DNAJB9 | F: GAGAGGATATCTGTGCCTGTCA | 810 |
|  | R: CTTAGTTAACTTCTTTCACACCCCA |  |
| RANBP17 | F: GCCAAAGAATCTATTTTCTATCAAAG | 807 |
|  | R: GCAATGCATTTTAATACTGTTTCAC |  |
| MYOC | F: TGGGAGGCTGAGGCAGGAGAATCAA | 810 |
|  | R: CAGGCGTGAGCCACCATGCCTG |  |
| UPP2 | F: CTAATTTCATTTCACTGTGGTCAC | 809 |
|  | R: AAAGATGTACCAGTCAAACTGTAAC |  |
| CNTN5 | F: AGGTCAGGAGATCGAGACCA | 810 |
|  | R: CTTGAACGCCAGGCCTCAAG |  |
| CCR5 | F: GGATTATCAAGTGTCAAGTCCAATC | 1054 |
|  | R: CAAGCCCACAGATATTTCCTG |  |
| gag | F: ATCAATGAGGAAGCTGCAG  R: CACATAATCCACCTATC |  |
| β-globin | F: ACACAACTGTGTTCACTAGC  R: TGGTCTCCTTAAACCTGTCTTG |  |

F:forward, R: reverse.
